# The Impact of Fluoride Pollution on Fungal Communities at the Watershed Scale: A Case Study of the Qingshui River, Ningxia

**DOI:** 10.3390/microorganisms13122733

**Published:** 2025-11-29

**Authors:** Zengfeng Zhao, Xiaocong Qiu, Juan Yin, Ruizhi Zhao, Cheng Ni

**Affiliations:** 1 School of Civil and Hydraulic Engineering, Ningxia University, Yinchuan 750021, China; zhaozf0517@163.com (Z.Z.); 12023131225@stu.nxu.edu.cn (C.N.); 2 School of Life Sciences, Ningxia University, Yinchuan 750021, China; 3 Ningxia Environment Monitoring Center, Yinchuan 750000, China; takuinugalaxy@sina.com

**Keywords:** fluorine, fungi, water-soil system, Ningxia, Qingshui River

## Abstract

This study systematically investigated the driving mechanisms and feedback effects of fluoride pollution gradients on fungal communities in water-soil systems, using the Qingshui River basin in Ningxia, China, as a case study. In 2022, 66 sets of samples, each comprising water, sediment, and riparian soil, were collected across three phases (May, July, December). High-throughput sequencing combined with fluoride speciation analysis revealed that fluoride pollution significantly reduced fungal alpha diversity (low-fluoride group > high-fluoride group I > high-fluoride group II), with aquatic habitats exhibiting the most sensitive response. Ascomycota and Chytridiomycota were identified as dominant fluoride-tolerant phyla, and *Ascobolus* and *Cladosporium* as representative tolerant genera. Fungi influenced fluoride speciation through mediating mineral weathering and organic matter metabolism; for instance, *Humicola* promoted fluoride immobilization, while *Archaeorhizomyces* participated in organic matter-bound fluoride (O.M.-F) metabolism. Fungi in sediments tended to promote the accumulation of residual fixed fluoride (Res-F), whereas those in riparian soils exhibited dual regulatory effects on the release of bioavailable fluoride (Ba-F). This research elucidates the succession patterns of fungal communities under fluoride pollution and their feedback mechanisms on fluoride biogeochemical cycling, offering a theoretical basis for ecological restoration in high-fluoride regions.

## 1. Introduction

Microorganisms are key drivers of biogeochemical cycles, playing crucial roles in regulating ecosystem functions, facilitating elemental migration, and driving mineral evolution [1]. The structure and function of microbial communities are not only regulated by environmental factors but are also profoundly influenced by complex interspecific interactions (e.g., competition, symbiosis, and predation) [2]. These interspecific relationships collectively form the basis of ecosystem response and resilience, determining a system’s adaptability to external disturbances [3,4]. For instance, fluoride pollution can lead to the disappearance of sensitive microbial taxa [5], creating ecological niches, reshaping interspecific interaction networks, and consequently driving community succession. Therefore, a comprehensive understanding of the mechanisms by which fluoride pollution affects microbial communities necessitates considering the ecological context of interspecific interactions.

Fluoride pollution, a global environmental problem [6,7], significantly impacts microbial communities due to its speciation and bioavailability in water-soil systems. However, most existing research has focused on microorganisms involved in nitrogen, phosphorus, iron, and sulfur cycling [8,9,10], while the effects of fluoride pollution on fungal communities and their potential role in fluoride speciation transformation remain poorly understood. This knowledge gap severely restricts our understanding of microbial response patterns in high-fluoride environments and impedes the development of microbial-based strategies for fluoride pollution remediation.

The Qingshui River, one of the most severely fluoride-polluted tributaries in the Ningxia section of the Yellow River [11], exhibits significant spatial heterogeneity in fluoride pollution. The formation of high-fluoride water is primarily controlled by evaporative concentration and sulfate mineral weathering, with minimal anthropogenic interference [12], making it an ideal area for studying the effects of fluoride pollution on microbial communities. Within the water-soil system of this watershed, fluoride exists in five forms: water-soluble fluoride (Ws-F), exchangeable fluoride (Ex-F), iron-manganese oxide-bound fluoride (Fe/Mn-F), organic matter-bound fluoride (O.M.-F), and residual fixed fluoride (Res-F) [13]. Among these, Ws-F and Ex-F are classified as bioavailable fluoride (Ba-F), posing a direct threat to regional ecological security. Studies have shown that aquatic biodiversity in the Qingshui River is significantly lower compared to other low-fluoride hydrological networks [14], and fluoride pollution further reduces bacterial diversity [13]. Fungi, as key decomposers and participants in elemental cycling in ecosystems [15,16], exhibit poorly understood community dynamics under high-fluoride stress.

The regulation of fungal communities by fluoride is complex. Low concentrations of fluoride may promote the growth of certain fungi, whereas high concentrations inhibit metabolic activity and reduce diversity [17]. However, some studies have also found that specific fungi can still proliferate in high-fluoride environments [18]. For example, *Aspergillus niger* can accelerate the weathering of fluorapatite to release fluoride ions even when its own growth is inhibited [19], indicating a nonlinear relationship between fluoride speciation and microbial responses. These contradictory findings suggest that the effects of fluoride pollution on fungal communities are not limited to simple toxic inhibition; instead, they may involve species-specific adaptation strategies and functional feedback mechanisms [20]. Nevertheless, a systematic investigation is still required to understand how fluoride pollution drives the succession of fungal communities in water-soil systems and whether community characteristics are specifically associated with fluoride speciation.

Based on these considerations, the present study proposes the following verifiable core hypotheses: (1) Fluoride pollution gradients will lead to predictable successional patterns in fungal community diversity and structure; (2) Indicator fungal taxa, either tolerant or sensitive to fluoride pollution, exist whose abundance changes are closely correlated with fluoride speciation; (3) Fungal communities feed back into fluoride speciation transformation processes through their metabolic activities. To test these hypotheses, this study focuses on the water-soil system of the Qingshui River basin. Combining high-throughput sequencing and fluoride speciation analysis, it primarily investigates the following: ① The succession patterns of fungal community diversity and structure along the fluoride pollution gradient; ② The dominant fungal taxa that are tolerant or sensitive to fluoride pollution; and ③ The coupling relationship between fungal community characteristics and fluoride speciation. This study aims to elucidate the succession mechanisms of fungal communities under fluoride pollution and their feedback effects on the biogeochemical cycling of fluoride, thereby providing a theoretical basis and technical support for ecological remediation in high-fluoride regions.

## 2. Materials and Methods

### 2.1. Overview of the Study Area

The Qingshui River basin is situated between 105°00′ and 107°07′ east longitude and 35°36′ and 37°37′ north latitude. It lies within a temperate semi-arid climate zone, with temperatures decreasing from north to south. The southern region is characterized by cooler and wetter conditions, while the northern region is warmer and drier [21]. The southern part belongs to the warm temperate semi-humid zone, the central part to the mid-temperate semi-arid zone, and the northern part to the mid-temperate arid zone [22].

### 2.2. Sample Collection and Pretreatment

**Figure 1:**
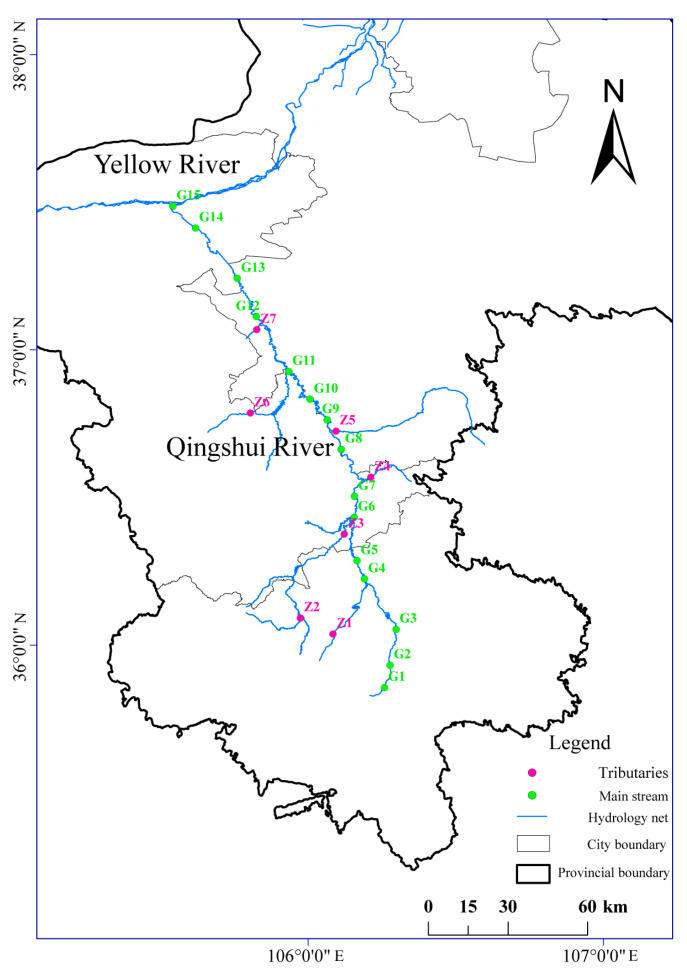
Distribution of sampling sites in the Qingshui River basin.

Based on the hydrological characteristics and tributary distribution of the Qingshui River, 22 sampling sites were established across the basin, including 15 on the main stream and 7 on the tributaries. The locations of these sites are shown in Figure 1. Sampling was carried out in May 2022 (normal period), July 2022 (wet season), and December 2022 (dry season). This sampling scheme encompasses different hydrological periods, aiming to minimize the effects of seasonal hydrological fluctuations and associated environmental factor variations on fungal community structure, thereby enabling a clearer assessment of the specific impacts of fluoride pollution. The collection of water and sediment samples was conducted in accordance with previously described methods [21,22,23]. Additionally, surface soil samples (0–20 cm) from the riparian and interstitial water in surface sediments were collected, with the latter extracted using a custom-made interstitial water sampler [22]. At each site, multiple subsamples were collected and thoroughly homogenized. Water, sediment, and riparian soil samples were each divided into two aliquots: one for fluoride analysis and the other for fungal ITS high-throughput sequencing. All samples were transported in insulated coolers and maintained at 4 °C. For fluoride analysis, water samples were stored at 4 °C and analyzed within 48 h, while sediment and soil samples were air-dried prior to analysis. For molecular analysis, samples designated for DNA extraction were immediately frozen at −80 °C upon return to the laboratory to preserve microbial community integrity until further processing.

### 2.3. Determination of Fluoride Speciation

Fluoride (F^−^) in surface water and interstitial water was measured using the ion-selective electrode method (GB/T 7484-87) [24]. T-F in sediment and soil was determined by the ion-selective electrode method (HJ 873-2017) [25]. The determination methods for the five fluoride speciation were referenced from the study by Zhao et al. [13], as follows:

(1) Ws-F: Weigh 5 g of soil sample, add 50.0 mL of deionized water, shake at room temperature for 30 min, allow to stand, and then centrifuge to collect the supernatant for analysis.
(2) Ex-F: Wash the centrifuged residue from step (1) twice, add 50 mL of 1 mol·L^−1^ NaAc solution, shake at room temperature for 1 h, centrifuge, and collect the supernatant for analysis.
(3) Fe/Mn-F: Wash the centrifuged residue from step (2) twice, add 50 mL of 0.5 mol·L^−1^ hydroxylamine hydrochloride solution, shake at room temperature for 1 h, centrifuge, and collect the supernatant for analysis.
(4) O.M.-F: Wash the centrifuged residue from step (3) twice, add 30% H_2_O_2_ and oxidize at room temperature for 2 h, then heat in a 90 °C water bath until the H_2_O_2_ is completely decomposed; add 3.2 mol·L^−1^ NH_4_Ac solution, shake at room temperature for 30 min, centrifuge, and collect the supernatant for analysis.
(5) Res-F: The content of Res-F is calculated by subtracting the sum of the other four fluoride speciation from the T-F.

All test solutions were analyzed using the ion-selective electrode method with the addition of TISAB buffer solution.

### 2.4. High-Throughput ITS Sequencing

Total DNA was extracted using the NucleoSpin 96 soi (MACHEREY-NAGEL, Dueren, Germany) according to the manufacturer’s instructions. The purity and concentration of the extracted DNA were assessed by agarose gel electrophoresis. An appropriate amount of DNA was transferred to a centrifuge tube and diluted with sterile water to a final concentration of 1 ng·μL^−1^. The diluted genomic DNA served as the template for PCR amplification of the ITS1 region of fungal rDNA, using primers ITS5-1737F (5′-GGAAGTAAAAGTCGTAACAAGG-3′) and ITS2-2043R (5′-GCTGCGTTCTTCATCGATGC-3′) [26]. PCR reactions were performed in a 15 μL volume containing High-Fidelity PCR Master Mix (New England Biolabs, Ipswich, MA, USA), 0.2 μM each of forward and reverse primers, and approximately 10 ng of template DNA. The thermal cycling protocol consisted of an initial denaturation at 98 °C for 1 min, followed by 30 cycles of denaturation at 98 °C for 10 s, annealing at 50 °C for 30 s, and extension at 72 °C for 30 s, with a final extension at 72 °C for 5 min.

PCR products were verified via 2% agarose gel electrophoresis and pooled in equimolar amounts. Target bands were excised and purified using the Qiagen Gel Extraction Kit (Qiagen, Hilden, Germany) after a second electrophoresis. Sequencing libraries were constructed using the NEBNext^®^ Ultra™ IIDNA Library Prep Kit (New England Biolabs, Ipswich, MA, USA), quantified by Qubit and qPCR, and qualified libraries were subjected to sequencing on the NovaSeq 6000 platform (Illumina, San Diego, CA, USA). Raw sequencing data underwent quality control and chimera removal. Denoising and filtering of sequences with an abundance of less than 5 were conducted using the DADA2 plugin in QIIME2 to generate amplicon sequence variants (ASVs) and feature tables [27]. Taxonomic assignment of ASVs was performed against the UNITE v8.2 database using the classify-sklearn module in QIIME2 to obtain taxonomic information. Sequencing and taxonomic annotations were carried out by Beijing Novogene Bioinformatics Technology Co., Ltd.(Beijing, China).

### 2.5. Stability Risk Assessment Model

Among the five existing speciation of fluoride, Ws-F and Ex-F are the most sensitive to environmental changes. Due to their weak binding strength, they are easily released under alkaline conditions, exhibiting rapid desorption and high bioavailability. This study employs the Stability risk assessment criteria (*SAC*) to evaluate the release risk of fluoride in sediments and riparian soils of the Qingshui River basin. This model is commonly used to assess the risk of fluoride release from sediments [28]. The stability risk assessment model is expressed in Equation (1).

(1) SAC=CeCt

In this equation, $C_{e}$ is the content of Ba-F, representing the sum Ws-F and Ex-F. $C_{t}$ is defined as the total extractable fluoride, including Ws-F, Ex-F, Fe/Mn-F, and O.M.-F. When *SAC* ≤ 1%, the system is classified as “extremely stable”; when 1% < *SAC* ≤ 10%, as “stable”; when 10% < *SAC* ≤ 30%, as “moderately stable”; when 30% < *SAC* ≤ 50%, as “unstable”; and when *SAC* > 50%, as “extremely unstable”.

### 2.6. Ecological Risk Assessment Model

This study employs the bioavailable fluoride-based potential ecological risk assessment model (${ER}_{bc}$) proposed by Xue et al. [29]. to evaluate the ecological risk of fluorides in sediments and riparian soils of the Qingshui River. The study by Zhao et al. has confirmed that the assessment results from this model more accurately reflect the ecological risk of fluorides in the Ningxia section of the Yellow River basin [11]. The calculation model is shown in Equation (2):

(2) ERbc=Tr×Cf−bc=Tr×Cbio−dCbio−0=Tr×(F1+F2)d(F1+F2)0

In the equation, $T_{r}$ represents the toxicity response factor, with a value of 1; $C_{f-bc}$ is the ratio of the measured Ba-F content to the reference value; $C_{bio-d}$ is the measured Ba-F content (mg·kg^−1^); $C_{bio-0}$ is the reference Ba-F content (mg·kg^−1^); $F_{1}$ is the Ws-F content determined by the sequential extraction method (mg·kg^−1^); $F_{2}$ is the Ex-F content determined by the sequential extraction method (mg·kg^−1^); $F_{1}$ + $F_{2}$ is the total Ba-F content (mg·kg^−1^); ${\left(F_{1}+F_{2}\right)}_{d}$ is the Ba-F content in the monitored sample (mg·kg^−1^); and ${\left(F_{1}+F_{2}\right)}_{0}$ is the Ba-F content in the reference sample (mg·kg^−1^). In this study, the average Ba-F content in sediments from the Ningxia section of the Yellow River basin (12.65 mg·kg^−1^) was selected as the reference value. When the value is <0.5, the risk is classified as “low”; when 0.5 ≤ value < 1, as “moderate”; when 1 ≤ value < 1.5, as “considerable”; when 1.5 ≤ value < 2, as “high”; and when ≥2, as “very high”.

### 2.7. Data Analysis and Visualization

Fungal α-diversity indices (Chao1, ASV, Pielou_e, and Shannon) were calculated using QIIME2 software (v 2023.5). Clustering analysis of species abundance for the top 35 genera and Spearman correlation analysis with fluoride speciation were performed, with all calculations and visualizations completed on the Novogene Cloud platform (https://magic.novogene.com/ (accessed on 1 November 2023)). Intergroup differences in fungal α-diversity indices were assessed on the Panosen Genes Cloud platform (https://www.genescloud.cn/chart/ChartOverview (accessed on 2 November 2023)) using the Kruskal–Wallis test and Dunn’s test for significance analysis.

The sampling site layout map was created using ArcGIS 10.6. Chord diagrams of the fungal community structure at the phylum level, fluoride release risk maps for sediments and soils, and ecological risk maps were all generated using Origin 2021. To minimize the confounding influence of other factors and highlight the specific impact of fluoride contamination, the 66 sample sets in this study were grouped based on surface water fluoride concentrations. In accordance with the “Surface Water Environmental Quality Standard (GB3838-2002) [30],” samples from the study area were stratified into three groups based on surface water F^−^ concentrations: a low-fluoride group (LF) (F^−^ < 1.0 mg/L, corresponding to surface water quality Class III or better; *n* = 20); a high-fluoride group I (HF I) (1.0 ≤ F^−^ < 1.5 mg/L, corresponding to surface water quality Classes IV–V; *n* = 35); and a high-fluoride group II (HF II) (F^−^ ≥ 1.5 mg/L, corresponding to surface water quality poorer than Class V; *n* = 11). For clarity in figures, WLF, SLF, and SOLF denote the low-fluoride groups for water, sediment, and soil, respectively. Similarly, WHF I, SHF I, and SOHF I represent the high-fluoride group I, and WHF II, SHF II, and SOHF II represent the high-fluoride group II for water, sediment, and soil, respectively.

## 3. Results and Analysis

### 3.1. Fluoride Content in the Water-Soil System of the Qingshui River

F^−^ concentrations in the surface water of the Qingshui River display a distinct gradient. The WLF has an average F^−^ concentration of 0.72 mg·L^−1^, while WHF I (1.23 mg·L^−1^) and WHF II (2.23 mg·L^−1^) are 1.71 and 3.10 times higher, respectively. In contrast, the increase in F^−^ concentration in interstitial water of surface sediments is more moderate, with values of 0.84 mg·L^−1^ in the low-fluoride group, 1.23 mg·L^−1^ in high-fluoride group I, and 1.66 mg·L^−1^ in high-fluoride group II, corresponding to increases of only 1.46 and 1.98 times, respectively [13]. The fluoride content in sediments and riparian soils is shown in Table 1. Although the total Ba-F content in sediments and riparian soils shows an increasing trend from the low-fluoride group to high-fluoride group I to high-fluoride group II, the magnitude of this increase is significantly lower than that observed for F^−^ concentrations in surface water. The gradient differences in total Ba-F across different habitats further validate the scientific and rational basis for habitat grouping based on F^−^ concentration gradients in surface water.

T-F is primarily composed of Res-F, and its distribution is controlled by geological background [31]. However, in the study area, the high-fluoride groups exhibit lower Res-F content compared to the low-fluoride group, which is speculated to be related to the transformation of Res-F speciation [13]. Riparian soils undergo frequent seasonal freeze–thaw cycles, where physical disintegration and chemical leaching act synergistically [32,33], potentially accelerating the conversion of Res-F into bioavailable speciation. Additionally, fluoride-bearing minerals, in riparian soils are continuously transported to the sediment system via surface runoff [34,35], which is another key reason why T-F and Res-F contents in soils are lower than those in sediments. Ws-F and Ex-F exist in a dynamic equilibrium; when a large amount of Ws-F is leached, other fluoride speciations are continuously transformed

### 3.2. Release Risk of Fluoride in Sediments and Riparian Soils of the Qingshui River

**Figure 2:**
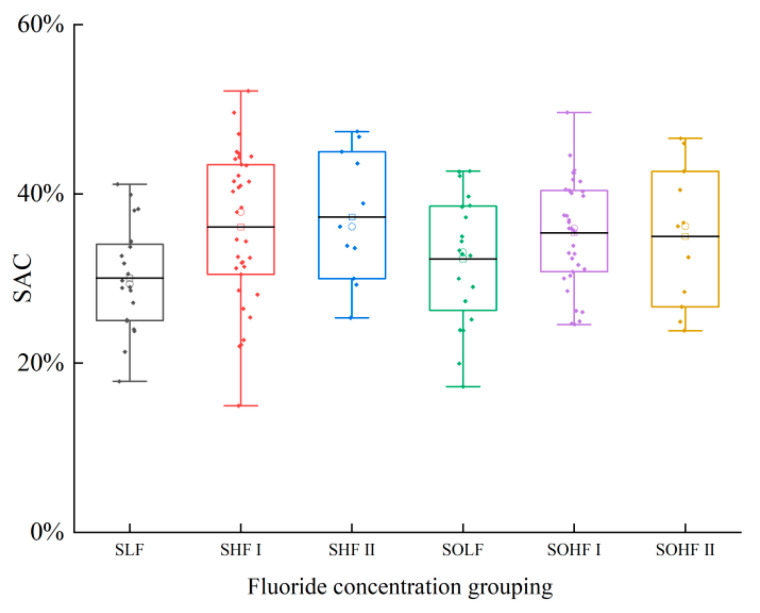
Risk of fluoride release from sediments and riparian soils in Qingshui River basin.

The SAC of fluoride in the sediments and riparian soils of Qingshui River is shown in Figure 2. The SAC of sediments ranges from 14.95% to 52.16%, with an average value of 34.44%, while the SAC of riparian soils ranges from 17.22% to 49.62%, with an average of 34.38%. All samples fall within a moderately stable to unstable state. Within sediments, SAC values varied among fluoride concentration groups. The highest SAC was observed in the SHF II at 37.25%, followed by the SHF I at 36.08%, and the lowest in the SLF at 30.03%. All these values consistently indicated an unstable state. This suggests that a substantial proportion of fluoride in sediments exists in an actively adsorbed speciation. Ws-F in sediments is a key driver of F^−^ concentration in surface water. Consequently, fluoride concentrations in the Qingshui River’s surface water are significantly influenced by sediment release. For riparian soils, SAC values across fluoride concentration groups were highest in the SOHF I at 35.38%, followed by the SOHF II at 34.97%, and the SOLF at 32.30%. This indicates that a considerable proportion of fluoride in riparian soils also exists in an actively adsorbed speciation. During runoff events, a substantial amount of Ws-F from riparian soils is transported into surface water. This leaching process results in lower Ws-F content in riparian soils compared to sediments.

### 3.3. Ecological Risk Assessment of Fluoride in Sediments and Riparian Soils Along the Qingshui River

**Figure 3:**
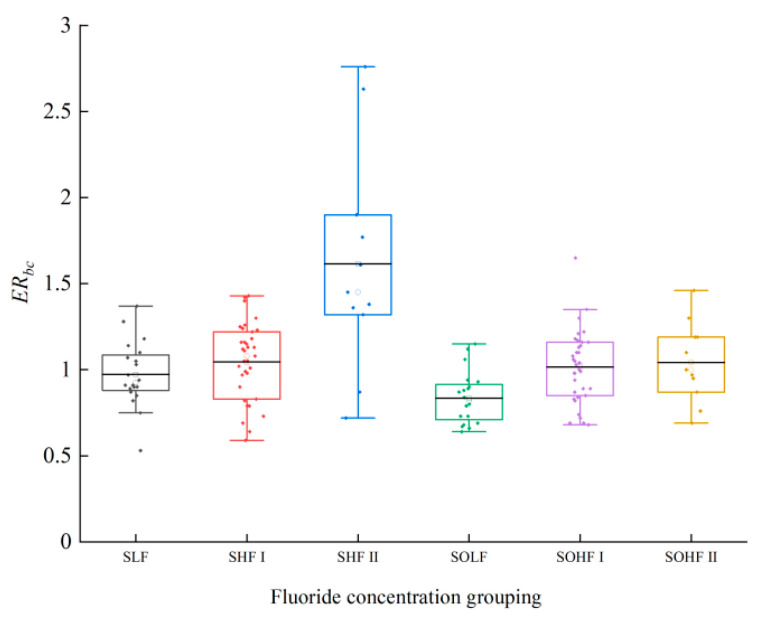
Ecological risk of fluoride in sediments and riparian soils of the Qingshui River.

The ecological risk of fluoride in the sediments and riparian soils of the Qingshui River is presented in Figure 3. In sediments, the average ${ER}_{bc}$ for the SLF was 0.97, indicating a moderate risk, 40% of samples were classified as considerable risk, with the remainder at moderate risk. For the SHF I, the average ${ER}_{bc}$ was 1.05, corresponding to a considerable risk; 62.86% of samples fell into the considerable risk category, while the rest exhibited moderate risk. The SHF II exhibited the highest average ${ER}_{bc}$ at 1.62, reflecting a very high risk. Within SHF II, 18.18% of samples were at extremely high risk, 27.27% at very high risk, 36.36% at considerable risk, and the remaining samples at moderate risk. For riparian soils, the average ${ER}_{bc}$ for the SOLF was 0.84, denoting a moderate risk; 15% of samples showed considerable risk, with the remainder at moderate risk. In the SOHF I, the average ${ER}_{bc}$ was 1.01, indicating a considerable risk; 2.86% of samples were at very high risk, 51.43% at considerable risk, and the remainder at moderate risk. The SOHF II also had an average ${ER}_{bc}$ of 1.04, signifying considerable risk, with 54.55% of samples at considerable risk and the remainder at moderate risk. Compared to the average ${ER}_{bc}$ of 1.00 for sediments from the Ningxia section of the Yellow River basin [36], the ecological risk of fluoride in both the sediments and riparian soils of the Qingshui River is relatively high, with the risk being particularly pronounced in sediments.

### 3.4. Venn of Fungal Communities in the Water-Soil System Under Different Levels of Fluoride Pollution

**Figure 4:**
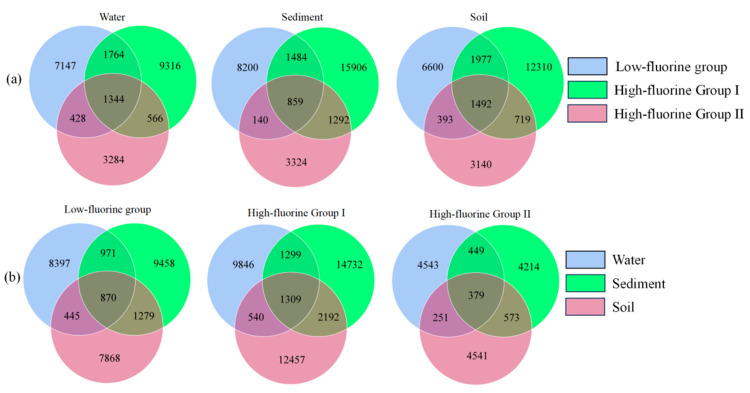
Venn of fungi under different grouping conditions. (**a**) presents a Venn diagram illustrating the shared and unique taxa across different fluoride concentration groups within a single habitat, and (**b**) depicts a Venn diagram showing the shared and unique taxa across different habitats within the same fluoride concentration group.

The Venn of fungal communities in the water-soil system under different levels of fluoride pollution in the Qingshui River basin are shown in Figure 4. Across different fluoride concentration groups within the same habitat, the number of ASVs followed the order: HF II < LF < HF I. Conversely, when comparing ASV counts across different habitats within the same fluoride concentration group, the following patterns were observed: for the LF, SOLF < WLF < SLF; for the HF I, WHF I < SOHF I < SHF I; and for the HF II, SHF II < WHF II < SOHF II. The smallest differences in ASV numbers among the three habitats were observed within the overall HF II. Considering the total unique ASVs across all three habitats for each fluoride concentration group, the order was HF I > LF > HF II. This suggests that the HF I may harbor a greater diversity of unique fungal species. Specifically, within the HF II, the water habitat (WHF II) exhibited a greater number of unique ASVs. Conversely, in both the LF and HF I, the sediment habitats (SLF and SHF I, respectively) harbored a higher number of unique ASVs.

### 3.5. Analysis of Differences in Fungal α-Diversity Indices in the Water-Soil System Under Different Levels of Fluoride Pollution

**Figure 5:**
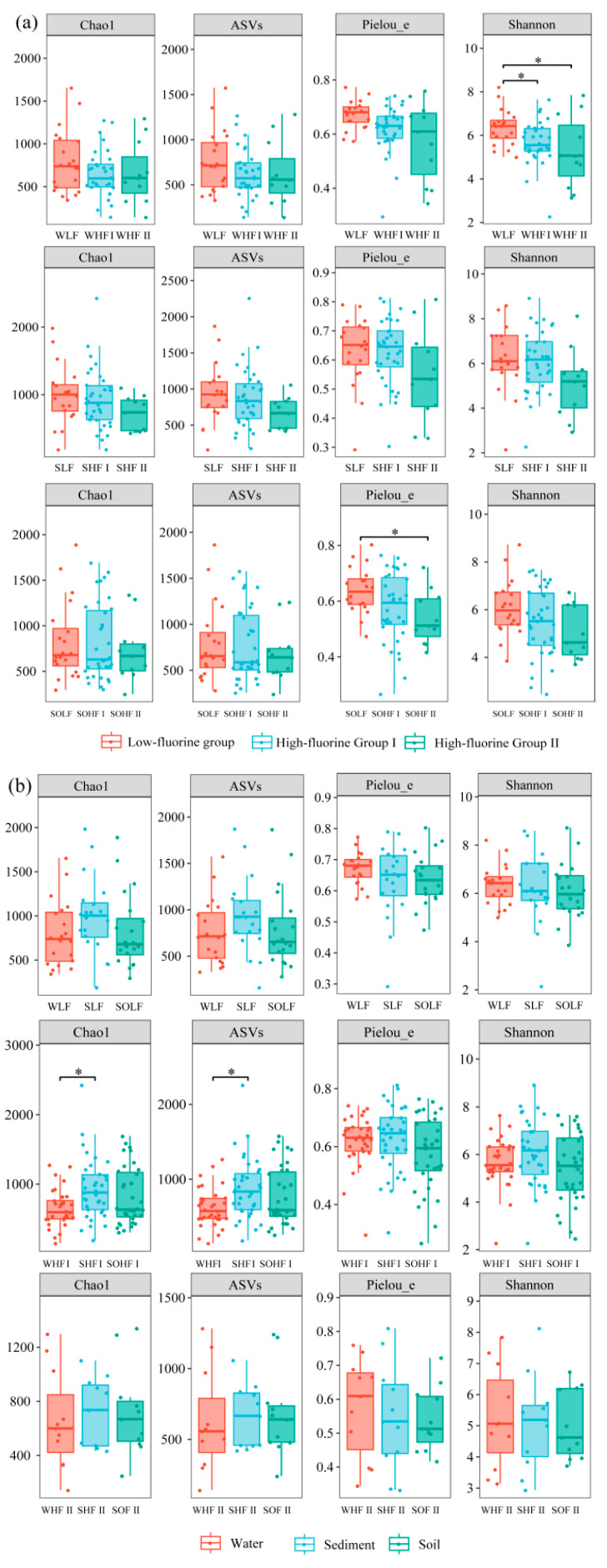
Fungal α-diversity indices under different grouping conditions (* indicates *p* < 0.05). (**a**) compares alpha diversity among different fluoride concentration groups within a single habitat, while (**b**) compares alpha diversity among different habitats within the same fluoride concentration group.

The fungal α-diversity indices (Chao1, ASV, Pielou_e, Shannon) for different fluoride concentration groups in the Qingshui River basin are presented in Figure 5. Overall, the α-diversity indices of fungi in both water and sediment exhibited a decreasing trend in the order: LF > HF I > HF II. In riparian soil, the Chao1 and ASV indices for fungi were lowest in the SOHF I, which may be associated with the highest Ws-F content observed in SOHF I. The Shannon index for fungi in water showed significant differences between the WLF and both the WHF I and WHF II (*p* < 0.05). Similarly, the Pielou_e index for fungi in riparian soil differed significantly between the SOLF and the SOHF II (*p* < 0.05). These results indicate that fluoride pollution may have altered fungal community structure, with a more pronounced impact on fungal communities in the water habitat compared to other environments. Within each fluoride concentration group, fungi in sediment generally displayed the highest α-diversity indices. This was particularly evident in the SHF I, where the Chao1 and ASV indices for fungi in sediment were significantly higher (*p* < 0.05) than those for fungi in water.

### 3.6. Fungal Community Structure at the Phylum Level in the Water-Soil System Under Different Levels of Fluoride Pollution

**Figure 6:**
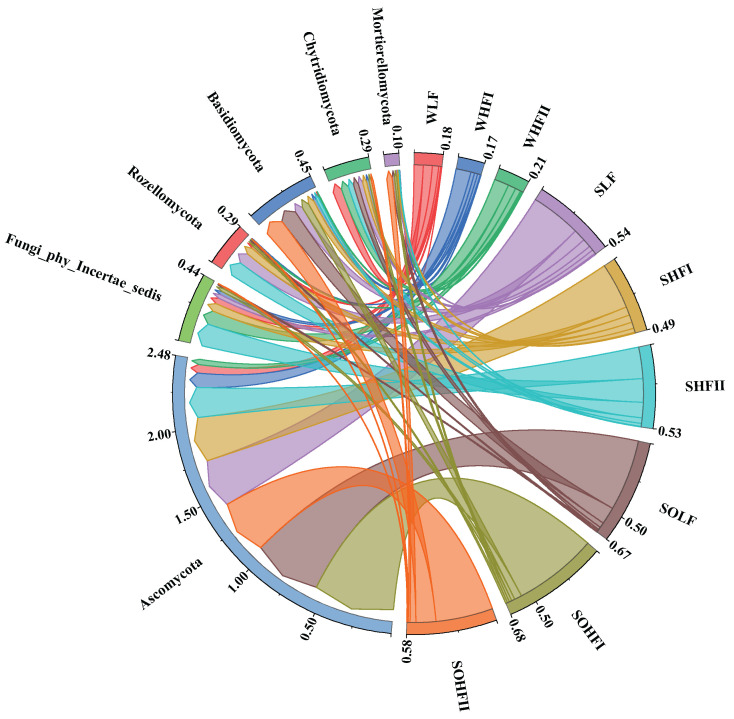
Chord diagram of fungal community structure at the phylum level under different fluoride concentration groups. Note: The designation ‘Fungi_phy_Incertae_sedis’ refers to a taxonomic group within the fungal kingdom whose classification at the phylum level remains uncertain.

The fungal community structure at the phylum level across different fluoride concentration groups in the Qingshui River basin is presented in Figure 6. Across the nine analyzed groups, the dominant phyla (with relative abundance >1%) were Ascomycota and Chytridiomycota. Ascomycota was highly abundant (relative abundance >5%) in all groups except for the WHF II. Chytridiomycota exhibited relatively high abundance in both the WLF and the WHF II. Fungi_phy_Incertae_sedis was relatively abundant in WHF II, as well as in the SHF I and the SHF II. Rozellomycota was relatively abundant across all three sediment fluoride concentration groups (SLF, SHF I, and SHF II). Basidiomycota, conversely, exhibited relatively high abundance in the SLF and in both the SOLF and the SOHF II.

### 3.7. Fungal Community Structure at the Genus Level in the Water-Soil System Under Different Levels of Fluoride Pollution

**Figure 7:**
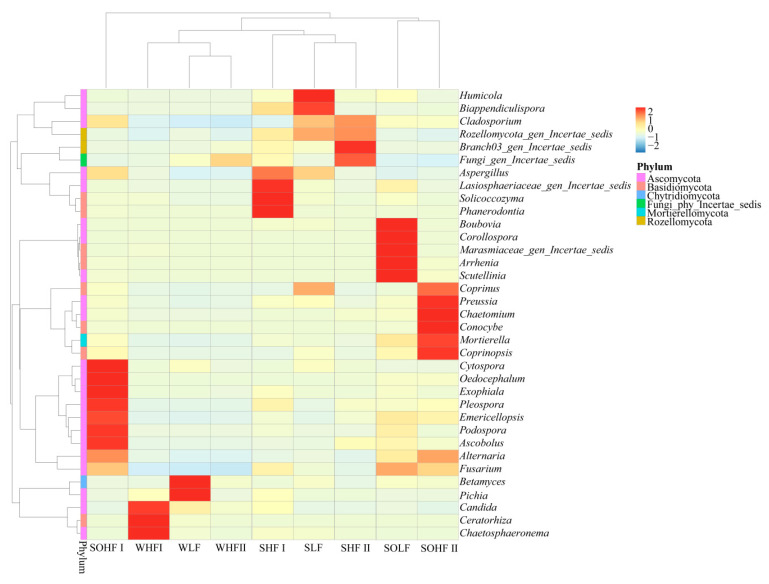
Cluster heatmap of fungal genus-level community structure under different fluoride concentration groupings. Note: The designations ‘*Fungi_gen_Incertae_sedis*’, ‘*Rozellomycota_gen_Incertae_sedis*’, ‘*Lasiosphaeriaceae_gen_Incertae_sedis*’, ‘*Branch03_gen_Incertae_sedis*’, and ‘*Marasmiaceae_gen_Incertae_sedis*’ refer to taxonomic groups whose classification at the genus level remains uncertain within the fungal kingdom, Rozellomycota, Lasiosphaeriaceae, Branch03, and Marasmiaceae, respectively.

The clustering heatmap of dominant genera in different fluoride concentration groups in the Qingshui River basin is shown in Figure 7. The water, sediment, and riparian soil in the HF I differ significantly from other fluoride concentration groups, exhibiting clear clustering differences across the different habitats. Among these, fungal communities in riparian soil exhibit the greatest differences at the genus level, while fungi in sediment and water show strong similarities. A total of thirty-five genera belong to six phyla. The dominant genera (relative abundance > 1%) include four in WLF, four in WHF I, and one in WHF II; eight in SLF, six in SHF I, and six in SHF II; and eleven in SOLF, thirteen in SOHF I, and nine in SOHF II.

Among the three water groups, *Fungi_gen_Incertae_sedis* is a shared dominant genus. *Rozellomycota_gen_Incertae_sedis*, belonging to the phylum Rozellomycota, *Pichia*, and *Betamyces* are unique dominant genera in WLF. *Chaetosphaeronema*, *Candida*, and *Ceratorhiza* are unique dominant genera in WHF I. WHF II has no unique dominant genera.

In the three sediment groups, *Fusarium*, *Fungi_gen_Incertae_sedis*, and *Rozellomycota_gen_Incertae_sedis* are shared dominant genera. *Alternaria* and *Coprinus* are unique dominant genera in SLF, while *Lasiosphaeriaceae_gen_Incertae_sedis* is a unique dominant genus in SHF I. *Ascobolus* and *Branch03_gen_Incertae_sedis* are unique dominant genera in SHF II. *Cladosporium* is a shared dominant genus in both the SLF and SHF II, while *Aspergillus* and *Biappendiculispora* are shared dominant genera in both SLF and SHF I.

In the three riparian soil groups, *Fusarium*, *Alternaria*, and *Cladosporium* are shared dominant genera. *Arrhenia*, *Boubovia*, *Scutellinia*, *Corollospora*, and *Marasmiaceae_gen_Incertae_sedis* are unique dominant genera in SOLF. *Podospora*, *Rozellomycota_gen_Incertae_sedis*, *Cytospora*, *Aspergillus*, *Oedocephalum*, *Exophiala*, *Pleospora*, and *Emericellopsis* are unique dominant genera in the SOHF I. *Conocybe*, *Coprinus*, *Preussia*, *Chaetomium*, and *Coprinopsis* are unique dominant genera in SOHF II. *Fungi_gen_Incertae_sedis* and *Ascobolus* are shared dominant genera in both SOLF and SOHF I, while *Mortierella* is a shared dominant genus in both SOLF and SOHF II.

### 3.8. The Relationship Between the Dominant Fungal Genera and the Fluoride Species in the Qingshui River

**Figure 8:**
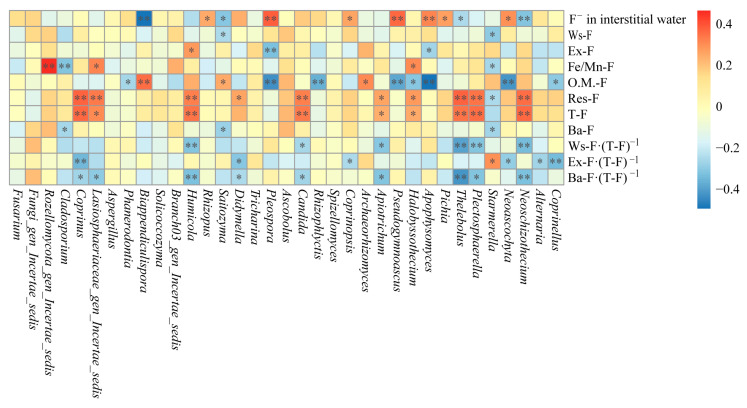
Correlation analysis between dominant fungi genera and fluorine species in sediments (* indicates *p* < 0.05; ** indicates *p* < 0.01).

The correlations between dominant fungal genera in sediments and different fluoride species are presented in Figure 8. Most dominant fungal genera in sediments show no significant correlation with Ba-F, primarily exhibit negative correlations with O.M.-F, and mainly positive correlations with T-F and Res-F. *Starmerella* and *Saitozyma* are significantly negatively correlated with Ws-F (*p* < 0.05). *Apophysomyces* and *Pleospora* are significantly (*p* < 0.05) and highly significantly (*p* < 0.01) negatively correlated with Ex-F, respectively, whereas *Humicola* is significantly positively correlated with Ex-F (*p* < 0.01). *Starmerella* and *Cladosporium* are significantly (*p* < 0.05) and highly significantly (*p* < 0.01) negatively correlated with Fe/Mn-F, respectively; in contrast, *Halobyssothecium*, *Lasiosphaeriaceae_gen_Incertae_sedis*, and *Rozellomycota_gen_Incertae_sedis* are significantly (*p* < 0.05) or highly significantly (*p* < 0.01) positively correlated with Fe/Mn-F. *Coprinellus*, *Neoascochyta*, *Apophysomyces*, *Halobyssothecium*, *Pseudogymnoascus*, *Rhizophlyctis*, *Pleospora*, and *Phanerodontia* show significant (*p* < 0.05) or highly significant (*p* < 0.01) negative correlations with O.M.-F, while *Archaeorhizomyces*, *Saitozyma*, and *Biappendiculispora* display significant (*p* < 0.05) or highly significant (*p* < 0.01) positive correlations with O.M.-F. *Neoascochyta*, *Plectosphaerella*, *Thelebolus*, *Halobyssothecium*, *Apiotrichum*, *Candida*, *Didymella*, *Humicola*, *Lasiosphaeriaceae_gen_Incertae_sedis*, and *Coprinus* exhibit significant (*p* < 0.05) or highly significant (*p* < 0.01) positive correlations with Res-F or T-F, whereas *Starmerella* is significantly negatively correlated with Res-F (*p* < 0.05).

In addition, *Neoschizothecium*, *Thelebolus*, *Saitozyma*, and *Biappendiculispora* show significant (*p* < 0.05) or highly significant (*p* < 0.01) negative correlations with F^−^ in interstitial water, whereas *Neoascochyta*, *Pichia*, *Apophysomyces*, *Pseudogymnoascus*, *Coprinopsis*, *Pleospora*, and *Rhizopus* exhibit significant (*p* < 0.05) or highly significant (*p* < 0.01) positive correlations with F^−^ in interstitial water. *Coprinellus*, *Alternaria*, *Neoschizothecium*, *Neoascochyta*, *Plectosphaerella*, *Thelebolus*, *Apiotrichum*, *Coprinopsis*, *Candida*, *Didymella*, *Saitozyma*, *Humicola*, *Lasiosphaeriaceae_gen_Incertae_sedis*, *Coprinus*, and *Cladosporium* demonstrate significant (*p* < 0.05) or highly significant (*p* < 0.01) negative correlations with Ba-F content, Ws-F proportion, Ex-F proportion, or Ba-F content proportion. *Starmerella* is significantly negatively correlated with Ba-F content (*p* < 0.05) and significantly positively correlated with Ex-F proportion (*p* < 0.05).

**Figure 9:**
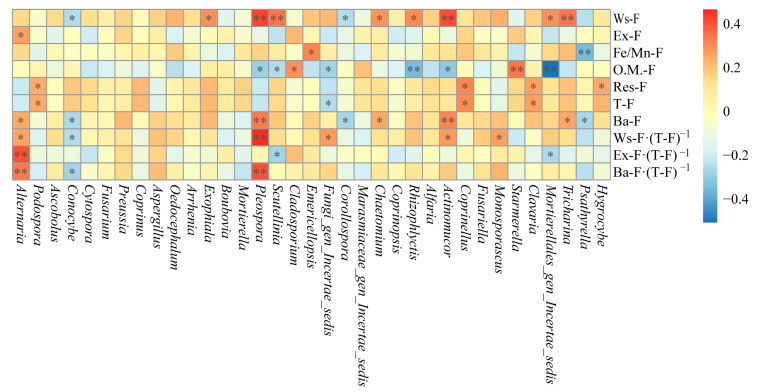
Correlation analysis between dominant fungi genera and fluorine species in riparian soil (* indicates *p* < 0.05; ** indicates *p* < 0.01). Note: The designation ‘*Mortierellales_gen_Incertae_sedis*’ refers to a taxonomic group whose classification at the genus level remains uncertain within the order Mortierellales.

As shown in Figure 9, compared to sediments, the riparian soil contains more dominant fungal genera significantly correlated with Ba-F, but fewer dominant genera significantly correlated with Res-F and T-F. *Tricharina*, *Actinomucor*, *Mortierellales_gen_Incertae_sedis*, *Rhizophlyctis*, *Chaetomium*, *Scutellinia*, *Pleospora*, and *Exophiala* exhibit significant (*p* < 0.05) or highly significant (*p* < 0.01) positive correlations with Ws-F, whereas *Corollospora* and *Conocybe* show significant (*p* < 0.05) negative correlations with Ws-F. *Alternaria* is significantly positively correlated with Ex-F (*p* < 0.05). *Psathyrella* and *Emericellopsis* are highly significantly positively correlated (*p* < 0.01) and significantly negatively correlated (*p* < 0.05) with Fe/Mn-F, respectively. *Mortierellales_gen_Incertae_sedis*, *Actinomucor*, *Rhizophlyctis*, *Fungi_gen_Incertae_sedis*, *Scutellinia*, and *Pleospora* exhibit significant (*p* < 0.05) or highly significant (*p* < 0.01) negative correlations with O.M.-F, whereas *Starmerella* and *Cladosporium* show highly significant (*p* < 0.01) and significant (*p* < 0.05) positive correlations with O.M.-F, respectively. *Hygrocybe*, *Clavaria*, *Coprinellus*, and *Podospora* display significant positive correlations (*p* < 0.05) with Res-F or T-F, while *Fungi_gen_Incertae_sedis* shows a significant negative correlation with T-F (*p* < 0.05).

In addition, *Tricharina*, *Monosporascus*, *Actinomucor*, *Chaetomium*, *Fungi_gen_Incertae_sedis*, *Pleospora*, and *Alternaria* show significant (*p* < 0.05) or highly significant (*p* < 0.01) positive correlations with Ba-F content, Ws-F proportion, Ex-F proportion, or Ba-F proportion, whereas *Psathyrella*, *Mortierellales_gen_Incertae_sedis*, *Corollospora*, *Scutellinia*, and *Conocybe* exhibit significant (*p* < 0.05) negative correlations with these same parameters.

## 4. Discussion

### 4.1. Response Patterns of Fungal Diversity and Community Structure Under Fluoride Pollution Gradient

With increasing fluoride pollution, fungal α-diversity generally shows a declining trend (LF > HF I > HF II), with water habitats exhibiting the highest sensitivity, as evidenced by the most pronounced changes in the Shannon index. The underlying mechanisms may include: (1) toxicity-driven directional selection, wherein high concentrations of fluoride exert strong selective pressure on sensitive taxa by compromising cell membrane integrity and inhibiting enzyme activity [20,37]; and (2) differential habitat exposure risks, compared to sediments and riparian soils, water lack buffering via adsorption by clay minerals or organic matter [38,39]. This results in fungi in water environments being directly exposed to more bioavailable fluoride, accelerating the process of community homogenization [18]. The synergistic effect of this “toxicity-exposure” dual stress ultimately leads to a simplified fungal community structure and a loss of functional redundancy in water systems.

Additionally, the Chao1 and ASV indices of fungi in riparian soil were lowest in SOHF I, which also had the highest Ws-F content, suggesting that Ws-F possesses greater biotoxicity. This pattern also provides a key insight for ecological restoration in fluoride-polluted areas, emphasizing the need to prioritize the regulation of fluoride species to reduce their bioavailability.

However, the Shannon and Pielou_e indices in WLF were both higher than those in SLF. This might be related to the higher F^−^ concentration in the interstitial water of SLF compared to the F^−^ concentration in WLF. Additionally, high concentrations of Cl^−^ in surface water may competitively reduce the influx of fluoride into the cytoplasm [12,37], thereby mitigating fluoride toxicity. The sediment system demonstrated a stronger ecological buffering capacity [40,41], with α-diversity higher than that of the riparian soil system. The low diversity characteristic of the riparian soil habitat may be attributed to the combined stress of high salinity and low moisture content in saline-alkali soils [42,43].

While this study primarily investigates the relationship between fungal community structure and fluoride gradients, we acknowledge that other parameters (e.g., pH, salinity, nutrient levels) may also influence microbial assembly [44,45,46]. For instance, high salinity in arid regions can exert osmotic stress [47] and shape fungal composition independently of fluoride. To minimize such confounding effects, we stratified samples based on fluoride levels and incorporated seasonal sampling to capture temporal heterogeneity [13]. Furthermore, previous studies in the same basin have demonstrated that fluoride is a significant driver of microbial community variation [22]. Nevertheless, future work should employ multivariate statistical models (e.g., RDA, PERMANOVA) to disentangle the relative contributions of multiple environmental drivers.

A key and nuanced finding was the pronounced number of unique ASVs in the HF I. This suggests that moderate fluoride stress acts as a distinct ecological filter, selecting for a specialized, transient assemblage. We propose that the HF I condition creates a transitional ecotone: the physiological cost of tolerance is not yet prohibitive, allowing for the persistence of some fluoride-sensitive taxa excluded from the HF II, while simultaneously providing a competitive advantage to early-successional, fluoride-tolerant specialists that are outcompeted by generalists in the LF. Alternatively, these unique ASVs may represent taxa indirectly promoted by fluoride-altered ecological interactions, such as the suppression of dominant competitors or predators in the LF, which releases previously rare taxa.

At the phylum level, the composition of dominant fungal phyla across different habitats and fluoride pollution gradients remains highly conserved, with widely distributed groups such as Ascomycota and Chytridiomycota predominating [48,49,50]. However, their relative abundances are synergistically regulated by both habitat characteristics and fluoride concentrations. Specifically: (1) Ecological adaptation and differentiation of broadly distributed phyla: the diversity of secretion systems in Ascomycota may support its colonization across various habitats [51,52,53]; Chytridiomycota may sustain high abundance in diverse environments through motile spore dispersal strategies, conferring niche occupancy advantages even under fluoride stress [54]. Additionally, Chytridiomycota possess a diverse array of enzymes capable of degrading complex organic matter, allowing them to exploit a wide range of nutritional resources and thereby enhancing their adaptability and abundance across various environments [55]. (2) Response specificity of fluoride-tolerant groups: the Fungi_phy_Incertae_sedis shows a significant increase in abundance in High Fluoride groups, yet only one of the 35 dominant genera belongs to this phylum, suggesting that fluoride tolerance may result from the combined resistance of multiple low-abundance taxa. (3) Functional genus succession as a driving mechanism: Rozellomycota and Basidiomycota maintain high niche occupancy across fluoride concentration gradients through genus-level functional differentiation. For example, *Arrhenia* (Basidiomycota) is more abundant in SOLF, while *Coprinopsis* predominates in SOHF II within the same zones, indicating fluoride-driven functional replacement among dominant genera.

At the fungal genus level, most groups display a dynamic response to the fluoride concentration gradient, with their abundance initially increasing and then decreasing, suggesting that long-term fluoride exposure may select for taxa with stage-specific tolerance. Cross-habitat analysis reveals that the number of dominant genera (relative abundance > 1%) is significantly higher in riparian soil systems than in water or sediments, highlighting pronounced community heterogeneity. *Fusarium*, a dominant genus found across multiple habitats, likely owes its tolerance to its adaptability to high-carbonate environments [56], a characteristic particularly relevant given that regional carbonate rock weathering is a major geochemical source of fluoride enrichment [12]. *Fusarium* may also contribute to the weathering and dissolution of carbonate minerals in the watershed. Within SHF and SOHF, genera such as *Ascobolus*, *Cladosporium*, and *Preussia* exhibit pronounced abundance, suggesting potential fluoride tolerance. Importantly, the competitive adsorption between phosphate and fluoride ions may affect fungal–environment interactions through biogeochemical processes [57]. For example, the consumption of orthophosphate by *Ascobolus* in the rhizosphere may indirectly enhance the capacity of riparian soils and sediments to immobilize fluoride ions [58].

### 4.2. The Influence of Dominant Fungi on the Transformation of Fluoride Species

The weathering of silicate minerals, a process significantly enhanced by fungal activity [59,60], is a key source of fluoride in the Qingshui River [12]. Fungal hyphae promote mineral dissolution through both biophysical penetration and the biochemical action of metabolic products [61,62]. This study expands the concept of “geomycology” by implicating carbonate weathering, potentially facilitated by genera like *Fusarium*, as another critical fluoride source within this watershed system.

Beyond weathering, fungi influence fluoride speciation through adsorption and mineralization. Fungal hyphal surfaces and their extracellular polymeric substances (EPS), rich in carboxyl and hydroxyl groups, provide nucleation sites for secondary mineral formation. The metabolic release of CO2 and organic ligands (e.g., oxalate) by fungi regulates dissolution-precipitation equilibria, driving biomineralization [63,64]. The adsorption of fluoride to EPS, a precursor to mineralization, parallels mechanisms well-documented for the immobilization of heavy metals and radionuclides like uranium and arsenic [65,66]. In sediments, dominant genera such as *Plectosphaerella*, *Thelebolus*, and *Humicola* showed significant positive correlations with T-F and Res-F, while correlating negatively with bioavailable fractions (Ws-F, Ex-F, Ba-F). This pattern strongly suggests these taxa promote the fixation of fluoride into stable mineral phases (e.g., fluorapatite), thereby reducing its environmental mobility and bioavailability.

The dynamic interplay between fungi, organic matter (OM), and fluoride further underscores their regulatory role. In sediments, genera like *Neoascochyta* and *Apophysomyces* were negatively correlated with F^−^ in interstitial water but positively with O.M.-F, indicating their potential role in sequestering fluoride into organic complexes. This process likely involves the adsorption of free F^−^ and metal-fluoride complexes (e.g., AlF_2_^+^, FeF_2_^+^) by functional groups in organic matter. Furthermore, complex microbial cascades link fluoride transformation to metal cycling; for instance, *Archaeorhizomyces* [67] may accelerate F^−^ release via OM decomposition, while the consequent release of Al^3+^ could enrich *Saitozyma* [68], demonstrating a tightly coupled system.

Although fluoride itself is not redox-active, its speciation is indirectly regulated by fungal metabolism through interactions with redox-sensitive elements (e.g., Fe, Mn). Fungi can alter fluoride mobility by decomposing organic matter or reducing metal oxides, processes that directly parallel their established roles in the biogeochemical cycling of arsenic and chromium [69,70].

In riparian soils, the fungal influence appears more bidirectional. While the overall contribution to mineralization may be weaker than in sediments, specific taxa like *Pleospora* and *Alternaria* may promote the release of Ba-F by decomposing Res-F. Conversely, taxa like *Actinomucor* and *Scutellinia*, by decomposing OM, may destabilize O.M.-F complexes. This reveals a dynamic balance where fungi can both immobilize and mobilize fluoride depending on the taxonomic and environmental context.

In conclusion, our findings position fungi as central architects in watershed-scale fluoride biogeochemistry. Their roles, spanning mineral weathering, EPS adsorption, organic matter transformation, and indirect redox mediation, draw clear parallels with the microbial cycling of other major contaminants. This suggests that established principles from geomicrobiology can inform future fluoride research, though the unique chemistry of fluoride demands tailored mechanistic studies to fully elucidate these complex interactions.

### 4.3. Limitations and Prospects of the Study

This study is the first to establish a coupled relationship between fluoride speciation and fungal communities at the watershed scale, addressing a significant gap in our understanding of fungal-driven fluoride biogeochemical cycling. However, due to the absence of a well-developed theoretical framework for the interaction mechanisms between fluoride speciation and microbes, the current conclusions are largely based on statistical correlations and lack direct evidence at the levels of metabolic pathways and functional genes. Future research should prioritize the development of cross-habitat, multi-interface microcosm models and the integration of metagenomics, transcriptomics, and other advanced techniques to specifically elucidate the molecular mechanisms underlying fungal-mediated transformations of fluoride speciation. Such efforts would facilitate the verification of functional response thresholds in fluoride-sensitive fungal taxa and clarify their ecological niche differentiation patterns, thus providing both theoretical foundations and technological strategies for the ecological remediation of fluoride pollution.

## 5. Conclusions

This study demonstrates that fluoride pollution acts as a key environmental filter, significantly reducing fungal α-diversity in the Qingshui River basin, with water habitats demonstrating the highest sensitivity. Fungal responses were characterized by the succession of stage-specific taxa, with Ascomycota and Chytridiomycota identified as key fluoride-tolerant groups; genera such as *Ascobolus* and *Cladosporium* are recognized as representative fluoride-tolerant taxa.

A central finding is the bidirectional role fungi play in the fluoride biogeochemical cycle. In sediments, genera like *Neoascochyta* promoted the fixation of Res-F, reducing its bioavailability. Conversely, in riparian soils, genera like *Pleospora* enhanced the release of Ba-F, underscoring the habitat-specific functional roles of fungi.

Looking forward, this work provides a foundational understanding of fungal-fluoride interactions at the watershed scale. To translate these correlative insights into mechanistic models and practical applications, future research should focus on:

(1) Employing multi-omics approaches (e.g., metagenomics, transcriptomics) to identify the key functional genes and pathways governing fluoride transformation.
(2) Developing fungal-based bioremediation strategies by screening and characterizing high-efficiency fluoride-immobilizing or releasing strains.
(3) Establishing cross-habitat microcosm experiments to quantitatively assess the contribution of specific fungal taxa to fluoride speciation under controlled conditions.

These efforts will be crucial for advancing the theoretical framework of fluoride biogeochemistry and informing effective ecological restoration strategies in high-fluoride regions.

## Figures and Tables

**Table 1:** Speciation characteristics of fluorides in sediments and riparian soils.

Habitat.	Grouping	Ws-F (mg·kg^−1^)	Ex-F (mg·kg^−1^)	Ba-F (mg·kg^−1^)	Fe/Mn-F (mg·kg^−1^)	O.M.-F (mg·kg^−1^)	Res-F (mg·kg^−1^)	T-F (mg·kg^−1^)
Sediment	SLF	9.21	3.10	12.31	10.36	19.39	522	564
	SHF I	10.34	2.89	13.23	9.50	16.18	427	466
	SHF II	17.69	2.75	20.44	9.55	26.08	439	495
Riparian soil	SOLF	7.64	2.92	10.56	7.00	16.43	464	498
	SOHF I	9.68	3.16	12.84	8.25	15.49	401	438
	SOHF II	9.05	4.15	13.15	7.17	17.82	414	452

## Data Availability

The data presented in this study are available on request from the corresponding author due to privacy concerns.
